# Increased number of brought-in-dead cases with COVID-19: is it due to poor health-seeking behaviour among the Zambian population?

**DOI:** 10.11604/pamj.2020.37.136.25967

**Published:** 2020-10-08

**Authors:** Misheck Chileshe, David Mulenga, Ruth Lindizyani Mfune, Tato Herbert Nyirenda, James Mwanza, Bright Mukanga, Steward Mudenda, Victor Daka

**Affiliations:** 1 Mary Begg Health Services, 56 Chintu Avenue, Northrise, P.O Box 72221, Ndola, Zambia,; 2 Copperbelt University, Michael Chilufya Sata School of Medicine, P.O Box 71769, Ndola, Zambia,; 3 University of Zambia, School of Veterinary Medicine, Department of Disease Control, P.O Box 32379, Lusaka, Zambia,; 4 The University of Zambia, School of Medicine, Department of Psychiatry, P.O Box 32379, Lusaka, Zambia,; 5 University of Zambia, School of Health Sciences, Department of Pharmacy, P.O. Box 50110, Lusaka, Zambia

**Keywords:** Coronavirus disease 2019, brought-in-dead, Zambia

## To the editors of the Pan African Medical Journal

**Figure 1:**
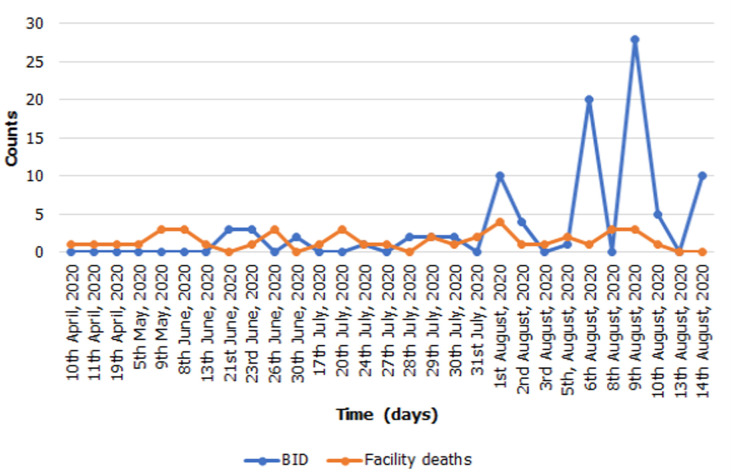
trends in the number of COVID-19 associated BIDs and facility deaths in Zambia

Since the first reported case on the 31^st^ December 2019 in Wuhan city, China, the current pandemic of the novel Coronavirus disease-2019 (COVID-19), caused by severe acute respiratory syndrome coronavirus - 2 (SARS-CoV-2), has resulted in over 889,256 deaths and over 27 million infections globally as of 23^rd^ August, 2020 [1,2]. Due to its rapid spread from China to many countries worldwide, COVID-19 was declared a global pandemic by the World Health Organization on 12^th^ March 2020 [1]. The spread of COVID-19 has been facilitated by increased human-to-human transmission and ignorance to observe the preventive measures [3]. Zambia recorded its first two cases of COVID-19 on 18^th^ March 2020. The first 28 cases in Zambia all had a history of travel to Europe or Asia. Three weeks into the outbreak, cases were noted among people without history of travel to COVID-19 endemic countries but were in contact with confirmed cases. There has since been an increase in local person-to-person transmission with increasing geographic spread. Cases have been reported in all ten provinces, with Western province being the last to record cases of COVID-19 [4]. We collected data for the period of five months, from April to August, 2020 from the official Zambia Ministry of Health website [5] and conducted a trend analysis to understand the frequency distribution of COVID-19 cases and associated deaths. Recently, Zambia has seen an increase in the number of COVID-19 cases and associated deaths. As of September 7, 2020, there were 12,836 confirmed cases of COVID-19 and a total of 295 deaths [5]. Of concern is that there has also been an increase in the number of COVID-19 brought-in-dead (BID) in the recent weeks with peak number being 28 BIDs on 9^th^ August, 2020 within 24-hours (Figure 1) [5]. Of the total 295 deaths, 214 were BIDs, indicating that 72.5% of the COVID-19 deaths are occurring in the community [5]. Here we raised pertinent questions regarding the health-seeking behaviour among the Zambian population amidst the COVID-19 pandemic.

Healthcare seeking behaviour (HSB) is defined as, “any action or inaction undertaken by individuals who perceive themselves to have a health problem or to be ill for the purpose of finding an appropriate remedy” [6]. Inappropriate HSB has been linked to worse health outcomes, increased morbidity and mortality, and poorer health statistics while early healthcare seeking has been reported to result in more favourable health outcomes [6]. Factors that define the healthcare seeking behaviour may be geographical, social, cultural and demographical. The use of healthcare services during COVID-19 pandemic may be determined by knowledge and information about the disease, perception of the illness, financial ability and social norms [6]. Stigma towards people infected with COVID-19 has also been reported as another contributing factor to poor HSB among people exhibiting suspected COVID-19 symptoms in Zambia [7]. Additionally, there has been a perception among the public that COVID-19 is not real and is fabricated leading to shunning of medical facilities despite having symptoms consistent with COVID-19 [7]. In Zambia, a number of studies have demonstrated poor health-seeking behaviour among the population. This has been reported among patients with tuberculosis, HIV, malaria, and even caregivers to under-five children. This has led to delayed hospital visitation because they perceived their illnesses to be common colds or malaria thereby opting to take home remedies or self-medication [8,9]. We believe this could be also the case of COVID-19 in which the symptoms mimic those of common colds and malaria, thereby making people to shun visiting healthcare facilities and practice self-medication. Furthermore, the prevalence of non-communicable diseases (underlying conditions) such as diabetes and hypertension among Zambian population may be higher than estimated and mainly undiagnosed. These conditions have been shown to worsen the course and prognosis of COVID-19 infection leading to increased mortality [10].

Current evidence indicates an increase in COVID-19 related brought-in-dead bodies in Zambia. With the increase in the number of cases making contact tracing more difficult, we hypothesize that this increase could be attributed to a poor healthcare-seeking behaviour among the Zambian population which may be due to a lack of knowledge about the disease, perception of the illness, stigma associated with the disease, readily available drug stores aiding self-medication as well as misconceptions about the severity of the disease due to the relative high proportion of recoveries with respect to cases. We recommend that studies on the perceptions of the Zambian population regarding COVID-19 be carried out to determine the drivers of the observed poor health seeking behaviour. We also recommend that there must be continuous and effective community sensitization and engagement programmes with regards to COVID-19 prevention and management. This information could be key in strengthening existing mitigation strategies against COVID-19 in Zambia.

## Conclusion

Current evidence indicates an increase in COVID-19 related brought-in-dead bodies in Zambia. This increase might be attributed to poor health-seeking behaviour among the Zambian population with COVID-19. We therefore recommend that studies on the perceptions of the Zambian population regarding COVID-19 be carried out to determine the drivers of the observed poor health seeking behaviour.
